# Supplementary material for: Does hepatectomy improve outcomes of breast cancer with liver metastasis? A nationwide analysis of real-world data in Taiwan
Source: PLoS One. 2022 Apr 21;17(4):e0266960. doi: 10.1371/journal.pone.0266960 (PMC9022801; doi:10.1371/journal.pone.0266960)
Supplement: Attachment — Submitted filename: Response to Reviewers.docx [file pone.0266960.s001.docx]

**Answer to Editor's comment**

**Editor:**

| Question | Answer |
| --- | --- |
| 1) Clearly identify that this analysis supports the hypothesis that liver resection is beneficial for a subset of patients with isolated liver metastases from breast cancer. | Thank you for your opinions.  We have adjusted our abstract and article to clarify our result. |
| 2) Please put together a summary table of literature with the number of patients/power from other studies so that a more complete understanding of the current literature may be appreciated. | Thank you for your opinions.  We have made the summary of table 3. |
| 3) Please address the comments made by reviewer 1 as best as possible. | Thank you for your opinions. We have addressed the comments from Reviewer 1. |

**Answer to Reviewers' comment**

**Reviewer #1**

| Question | Answer |
| --- | --- |
| Reviewer #1: The authors address an important question of liver resection in BCLM. However, the study is lacking in strength. The authors acknowledge this deficiency but persist to draw major conclusion. The discussion merely is review of literature and doesn't discuss the study findings being validated by other similar studies. no data on adj therapies or on liver ablative therapies in non resection group. no data on extent of liver disease, type of resection, tumor biology, adjuvant or neoadj therapies. While the authors acknowledge the limitations, they still draw major conclusion that liver resection is beneficial.  While this may be true, the data is lacking and insufficient to rule out confounding factors. | Thank you for your valuable opinions.  We have made the discussion between our study and those reviewed literates of the adjuvant therapy, and other therapies for treatment of liver mets from breast cancer, address the limitation in discussion section and marked.  Also, we have adjusted our conclusion and marked.  **Conclusion in abstract:**  These findings indicate that liver resection may offer better survival benefit in patients with breast cancer who develop new liver metastases post mastectomy.  **Conclusion in Article:** The present findings demonstrate that liver resection may offer survival benefit in patients with breast cancer who develop hepatic metastases post mastectomy. Based on our findings, liver resection could be considered as a treatment option for improving the overall survival of such patients. Further studies are required to validate the effectiveness of multidisciplinary approach and personalized treatment for BCLM. |
